# Diversity of Ants and Termites of the Botanical Garden of the University of Lomé, Togo

**DOI:** 10.3390/insects10070218

**Published:** 2019-07-23

**Authors:** Boris Dodji Kasseney, Titati Bassouo N’tie, Yaovi Nuto, Dekoninck Wouter, Kolo Yeo, Isabelle Adolé Glitho

**Affiliations:** 1 Laboratoire d’Entomologie Appliquée, Département de Zoologie et de Biologie Animale, Université de Lomé, 01 BP 1515, Lomé 01, Lome 151, Togo; 2 RBINS Scientific Service Heritage/O.D. Taxonomy and Phylogeny, Curator Entomology Collections, Vautierstraat 29, 1000 Brussels, Belgium; 3 Université Nangui Abrogoua, 02 BP 801 Abidjan 02, Abidjan, Côte d’Ivoire

**Keywords:** Biodiversity, Ants, Termites, former agricultural land, habitats restoration

## Abstract

Ants and termites are used as bioindicators in many ecosystems. Little knowledge is available about them in Togo, especially ants. This study aimed to find out how ants and termites could be used to assess the restoration of former agricultural land. These insect groups were sampled within six transects of 50 × 2 m^2^ (using pitfall traps, monoliths, baits for ants and hand sampling for termites) in two consecutive habitats: open area (grassland) and covered area (an artificial forest). Seventeen termite species and 43 ant species were collected. Seven ant species were specific to the covered area against four for the open area, while four unshared species of termite were found in the open area against three in the covered area. The presence of unshared species was linked to vegetation, as *Trinervitermes* (Holmgren, 1912), a grass feeding termite, was solely found in open area. Also, for some ant species like *Cataulacus traegaordhi* (Santschi, 1914), *Crematogaster* (Lund, 1831) species, *Oecophylla* *longinoda* (Latreille, 1802) and *Tetraponera mocquerysi* (Brown, 1960), all arboreal species, vegetation was a determining factor for their presence. The occurrence of these species together with *Basidentitermes mactus* (Sjöstedt, 1911), *Strumigenys bernardi* (Brown, 1960) and *S. sistrura* (Bolton, 1983), suggest a more advanced level of restoration of the covered area.

## 1. Introduction

For decades, the world has been facing a remarkable loss of species including terrestrial aquatic organisms [1,2,3,4]. The rate of this irreversible loss of biological diversity is getting more and more important according to recent studies [5,6,7]. Global warming is pointed out to be one of the main causes [4,7]. Global warming, however, appears to be linked to many factors directly or indirectly imputable to human activities such as farming, mining, urbanization and industries which impact species through habitat destruction and pollution [8,9]. As a response to these disturbances certain species may disappear, some may shift their ranges towards sites with better living conditions, while others are condemned to undergo some adaptations (physiological or morphological) allowing them to maintain in place [9]. Sometimes, the native species are just replaced by aliens equipped to face the harsh conditions. 

Efforts to reduce global warming, and hence the loss of biodiversity, include the restoration of degraded ecosystems [10,11,12,13] through afforestation [14,15,16,17,18]. In urban areas, especially in the tropics, where the establishment or enlargement of cities is destroying original habitats, the creation of green spaces, parks or botanical gardens is important to residents. Indeed, forested parks provide many benefits to city dwellers such as pleasant landscape and recreational space that they visit for clean air and tranquility [19]. Beside these benefits to residents, forested parks also serve as refuge for many critical species [20]. Such management can not only lead to the return of former inhabiting species but also to the establishment of rare and threatened ones [21]. Some of these species (including ants and termites) are very sensitive to the quality of their surrounding habitat. Their diversity as well as their responses toward habitat changes overlap with those of sympatric organism [22,23,24]. Species that react quickly to changing environments and that are used to detect these changes are called bioindicators [24]. Among the commonly used bioindicator taxa, animals represent 50%. Of this 50%, invertebrates occupy 70% [24]. Indeed, a huge number of invertebrate species are being used as bioindicators including beetles [25,26,27,28], butterflies [28,29,30], ants [31,32,33] as well as termites [34]. Ants (Formicidae) have been used to monitor changes in various ecosystems such as grassland [33], mountains [35], rainforest [36], tropical forest [37], arboreal plantation such as mango [38], cocoa [39], teak [40]. Beside ants, termites are also candidate as ecosystem monitoring tool [34,41,42] and in specific cases both can be quite good bioindicators. Hence, they were used in our study, to check the effect of reforestation on the reconstitution of a representative fauna. The specific aims of our study were: (i) to evaluate the diversity of ant and termite fauna within two restored areas (one afforested and the other left open with herbaceous plants) as well as their repartition and specificity across these habitats; and (ii) to delineate potential bioindicator species for those two groups.

## 2. Materials and Methods 

### 2.1. Study Site

**Figure 1:**
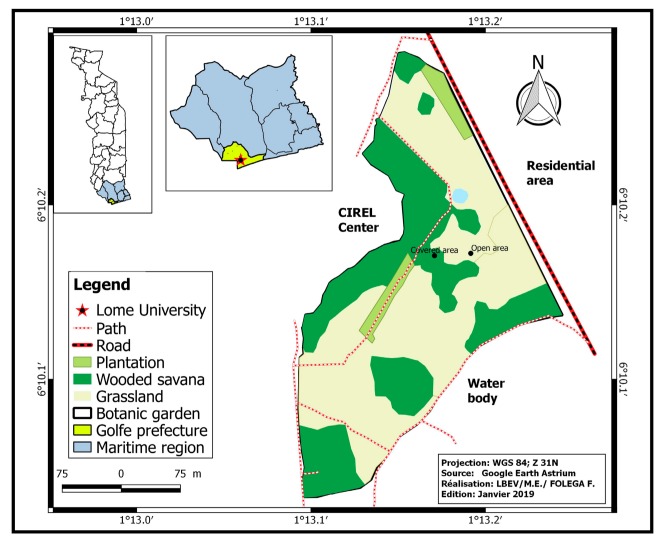
Map of the study site.

Ants and termites were sampled within the botanical garden of the University of Lomé (Figure 1) between October and November 2016. Lomé, the capital city of Togo is located in the southern part (golfe prefecture) of the country. There are two main rainy seasons (April to July and September to October) separated by two dry seasons (November to March and August). However, because of climate fluctuation, the length of these seasons varies across years. The small rainy season (September to October) and small dry season (August) are sometimes overlapping. The mean temperature recorded during the study period was 26.43 °C and precipitation in that period was 84 mm.

The study area (with around 3 ha) was previously cultivated land (the whole campus) up to 1988, when the garden was created. From that year, it was divided into two main parts: open area (with almost no trees) and an area planted with exotic plants and native ones (Table 1). The planted area is actually a thirty-year old artificial forest with some grassy patches near the edges and small lianas growing in the rainy season. Although they have a common border, the covered area (artificial forest) contrasts with the open area where the grass *Heteropogon contortus* (L.) P. Beauv. Ex Roem. and Shult (Poaceae) is the main plant encountered except one plant of *Azadirachta indica* A. Juss (Meliaceae) (Table 1). We considered these two areas as different habitats and called them here: the open area and the covered area. Although the grasses underneath and the growing liana of the covered area as well as the grass of open area are regularly cut once a year, we regarded both areas as having been conserved and managed since the cessation of agricultural activity in 1988.

### 2.2. Sampling 

**Figure 2:**
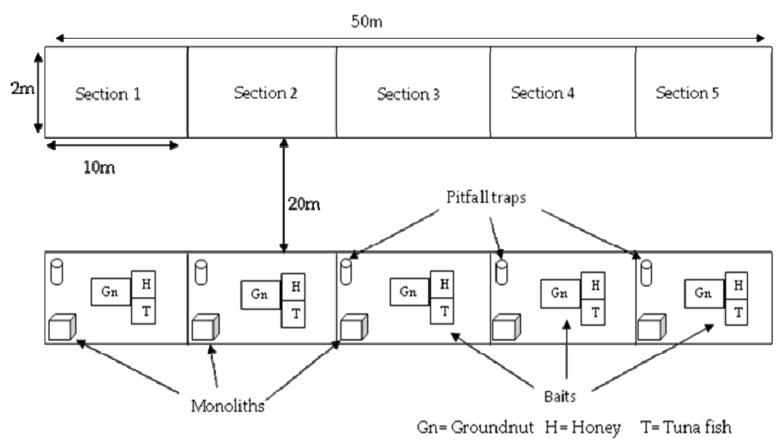
Schema of transect with different sampling methods.

Ants and termites were sampled within the frame of six different transects in the two studied habitat types. Three transects of 50 m length and 2 m width were laid in each habitat. Two consecutive transects were separated at least by a distance of 20 m. (Figure 2)

#### 2.2.1. Ants

Ants were collected within the same transects as termites but at least two weeks before the sampling of termites. Three standards methods [43] were used to collect ants: soil monolith, pitfall trap and bait traps (with honey, groundnut and tuna fish). Each 50 m transect was divided into five sections of 10 × 2 m^2^ that represented a sampling unit.

#### 2.2.2. Pitfall Traps

In each section, one open cup of 8.5 cm diameter and 14.5 cm depth was placed in the ground. The lip of the cups was flush with soil or leaf litter. In this way ants could easily fall inside the trap without any effort of climbing or turning around. In order to keep ants inside the trap, ethanol 70% was poured in each cup up to 25% of their total volume. The opening of each cup was protected with fallen leaves and grasses and the area surrounding the trap was reconstituted as near as possible to its original form. Two consecutive traps were 10 m apart from each other. The traps were removed after 24 h and collected ants were conserved in 90% ethanol in vials. For each transect, five pitfall traps were checked.

#### 2.2.3. Soil Monoliths

Blocs of soil of 30 × 30 × 15 cm^3^, dug out in each section opposite to pitfall traps were analyzed. Like pitfall traps, two consecutive soil monoliths were 10 m apart. The soil of the blocs was poured inside flat containers and carefully checked for ants. Encountered ants were collected and preserved in 90% ethanol in vials. For each transect, five monoliths were checked. 

#### 2.2.4. Bait Trap

Three different baits (honey, crushed groundnuts and tuna fish) were used. One table spoon of each bait substrate was put in the middle of a single piece of white paper (each single piece of paper supporting only one kind of substrate). Each set (substrate and supporting paper) constituted a bait trap. The three obtained bait traps were randomly placed in a triangle (but separated one from another by 5 cm) in the middle of each section. Two consecutive bait trap sets were separated by 10 m length. All the baits were removed after one hour. Attracted ants were collected and conserved in 90% ethanol in vials. 

#### 2.2.5. Direct Sampling

Unlike the above three described methods, the direct sampling was not carried out within the frame of the delimited transects. Through this method, ants were hand-collected randomly through the whole study area. Sampled ants were conserved in 90% ethanol in vials.

#### 2.2.6. Termites

We used a modified version of the protocol described by Jonnes and Eggleton [44] to sample termites. The same transect design used to sample ants was also used for termites. Each transect was divided into 10 small sections of 5 m length and 2 m width. Within the frame of each small section, we looked for termites above ground, in their nest, wood logs, and twigs; on living and dead trees, for 15 min. After active hand searching, soil-dwelling termites of the same section were sampled through eight soil scrapes (15 × 15 cm, 10 cm depth). Collected termites were conserved in 90% ethanol in vials and kept in the Laboratory of Applied Entomology for further processing. 

### 2.3. Identifications

#### 2.3.1. Ants

Sampled ants were sorted and grouped by morphospecies. Three to five specimens per morpho-species were mounted for identification. The identification was carried out to species level using the online keys of the “Ants Africa” website [45] as well as the published keys provided by Bolton [46] and Fisher and Bolton [47] and also using the code established for ant fauna of Côte d’Ivoire by Yeo Kolo (personal data).

#### 2.3.2. Termites

The collected termites were sorted and grouped by species or morphospecies (for ambiguous species). The identification to species level was based on the external morphological traits of soldiers and, if necessary, workers, using published keys [48,49,50,51,52,53]. Following identification, the specimens of termites and ants were preserved in the Laboratory of Applied Sciences of the University of Lomé (Togo).

### 2.4. Data Analysis

#### 2.4.1. Ants

We considered each single transect as sampling unit and as a replica for our data analyses. Because ants as well as termites are both social insects and the occurrence of a single individual indicates the presence of a whole colony nearby, we used the occurrence (presence or absence data) instead of absolute number of individuals. As the main objective of this study was to estimate the specific richness of ants and termites that inhabit the study site, we pooled the data obtained throughout the pitfall traps, monolith and bait traps. The data obtained by direct sampling were not pooled, however, because the ants were sampled not only within the time frame of the delimited transect but mostly across both habitats or between two consecutive transects.

To see how strong our estimates of species richness were, we computed several estimates of total species such as Chao2, Jacknife 1, Jacknife 2 and Bootstrap. We then calculated some alpha diversity index namely, the total observed richness (Sobs), Simpson index of diversity (1-D), Shannon–Wiener index, evenness, equitability for each single habitat with Past software [54]. The values of Simpson index of diversity (1-D) range between 0 and 1. A value close to 1 indicates greater sample diversity. The diversity t test was performed between the value of Shannon and Simpson index (D) of the two habitats with the same software. Finally Estimates 9 software [55] was used to compute shared species between the two habitats: shared observed species, Estimated number of species shared by the two habitats and Classic Jaccard index.

#### 2.4.2. Termites

The same statistical analyses were performed with the termite samples. However, unlike ants we do not need to pool any data as termites were sampled using a single method. 

## 3. Results

### 3.1. Ants

A total of 43 ant species belonging to 19 genera and six subfamilies (Table 2) were identified throughout 229 occurrences. None of the different used methods yielded the total number of 43 species. Direct sampling, Monolith and Pitfall traps seemed to be more effective in ant sampling respectively with 21; 26 and 28 ant species (Figure 1) than baits. Baits (respectively Honey, Groundnut and Tuna) yielded only three species (for the first two baits) and two (for the last one) species belonging to Formicinae (*Lepisiota* sp.1) and Myrmicinae (*Pheidole senilifrons* (Wheeler, 1922), *Pheidole tenuinodis* (Mayr, 1901) and *Trichomyrmex oscaris* (Forel, 1894)). While two species (*Ph. tenuinodis* and *T. oscaris*) were collected using all six sampling methods, three species were found only by direct sampling: *Camponotus* sp.1 (Formicinae), *Camponotus vividus* (Forel, 1913) and *Pheidole excellens* (Mayr, 1862). The four species of the Ponerinae, *Brachyponera sennaarensis* (Mayr, 1862) and the three *Hypoponera* species were sampled only by monliths (Table 2). 

Among the identified subfamilies, Myrmicinae (22 species) and Formicinae (13 species) were more represented with respectively 51.16% and 30.23% of the total occurrences (Table 2). Less than five species were found to belong to each of the other sub-families: Ponerinae (four species), Dolichoderinae (two species), Dorylinae and Pseudomyrmicinae each with only one species (Table 2). The most occurred species during our study period in both two habitats (open and covered areas) was *Ph. tenuinodis* (67 occurrences) followed by *Ph.senilifrons* (37 occurrences) and *Pheidole impressifrons* (Wasmann, 1905) (15 occurrences). The other species were less observed during the same period (incidences between 1 and 12) (Table 2).

The data from all the sampling methods except direct sampling yielded a total of 40 species. Nevertheless, the estimators of total species (Chao 2 (52.54 ± 6.87), Jacknife 1 (53.5 ± 2.5) and Bootstrap (46.75)) were all greater than our total observed species (Sobs = 40) suggesting that this value was not high enough (Table 3).

#### 3.1.1. Specificity of the Two Habitats 

In 128 occurrences, 24 species were found in open area (as mentioned above) while 29 species from 101 occurrences were found in covered area. Simpson index of diversity (1-D) for both open area (0.861) and covered area (0.865) was close to 1 indicating a great diversity for both areas (Table 4). This pattern of great diversity was confirmed by Shannon Wiener index: 2,48 and 2.63 respectively for open and covered area. However, no significant difference for Shannon index was found between these two areas (*t* = −0.91, df = 206, *p*(same) = 0.36) nor for Simpson index (*t* = 013, df = 204.31, *p*(same) = 0.89) (Table 5).

#### 3.1.2. Shared Species

Among the nineteen identified genera, 10 were common to both habitats, whereas four were collected specifically in the open area (*Brachyponera* (Emery, 1900), *Paratrechina* (Motschoulsky, 1863), *Nylanderia* (Emery, 1906) and *Trichomyrmex* (Mayr, 1865)) and five were sampled specifically in the covered area: *Cataulacus* (Smith, F., 1853), *Crematogaster* (Lund, 1831), *Oecophylla* (Smith, F., 1860), *Strumigenys* and *Tetraponera* (Smith, F., 1852). 

Fifteen genera were collected in the covered area and 14 in the open area with the different methods except direct sampling. Both habitats shared 12 species (Table 6). Jaccard (32.5%) and Sørensen classic (49%) showed that the two habitats shared less than half of the encountered species (Table 6). These shared species included *Tapinoma lugubre*, *Dorylus* sp.1, *Cardiocondyla emeryi*, *Monomorium* sp.1, *Camponotus acvapimensis*, *C. sericeus*, *Lepisiota guineensis*, *L. laevis* and four species of *Pheidole* (*Ph. impressifrons*, *Ph. senilifrons*, *Ph. tenuinodis* and *Pheidole* sp1). Species belonging to genera sampled solely from the open area included *Brachyponera sennaarensis*, *Paratrechina longicornis*, *Nylanderia boltoni* and *Trichomyrmex oscaris*. Those belonging to genera found only in the covered area included *Cataulacus traegaordhi*, *Crematogaster* (sp.1 and sp.2), *Oecophylla longinoda*, *Strumigenys bernardi*, *S. sistrura* and *Tetraponera mocquerysi*. 

### 3.2. Termites

Throughout 263 incidences, 17 termite species belonging to 10 genera and five subfamilies were collected (Table 7). The Macrotermitinae subfamily had the highest number of species (7) followed by the Nasutitermitinae (4), Termitinae (3) and Apicotermitinae (2). For the Cubitermitinae subfamily only one species was collected. The Fungus growing termites which all belong to Macrotermitinae was the most represented group followed by Grass feeding termites that exclusively belong to the Nasutitermitinae subfamily and that were represented by the four species of *Trinervitermes*. Soil and wood feeding groups were represented by three species each.

The value of total observed species (17) (see Table 8) was lower than that of chao2 (17.96 ± 1.28), but slightly lower than the values of Bootstrap (18.75) and Jacknife 1 (20.5 ± 0.5). This indicated that the total observed species was not high enough.

#### 3.2.1. Specificity of the Two Habitats

In the open area, 14 termite species were sampled in 154 occurrences against 13 species in 109 occurrences for covered area (Table 9). Simpson index of diversity (1-D), 0.897 and 0.863 respectively for open and covered areas were close to the maximum value (1). The diversity t test of both Shannon and Simpson index (Table 10) showed that open area was more diversified compared to covered area (*t* = 1.995, df = 197.2 and *p*(same) = 0.0048 for Shannon index; *t* = −1.98, df = 155.2 and *p*(same) = 0.0049 for Simpson index).

#### 3.2.2. Shared Species

Ten out of 17 species were shared by the two habitats (Table 11). The value of shared species was not equal to that of Chao shared estimates (10.67). Jaccard (58.8%) and Sørensen classic (74%) showed that these habitats shared between 50 and 75 % of encountered species. The unshared species (Table 11) included *Amitermes evuncifer*, *Trinervitermes geminatus*, *T. togoensis* and *Trivervitermes sp* (solely encountered in open area), *Microtermes tumodiensis*, *Microcerotermes* sp.2 and *Basidentitermes mactus* (sampled only in covered area).

## 4. Discussion

### 4.1. Ants

Ant sampling in both habitats yielded a total of 43 species and 20 ant species were sampled throughout direct sampling (21), monoliths (26) and pit fall trap (28). Direct sampling appears to yield good results to understand ant diversity as was the case in mainland savannas of the Venezuelan Llanos [56]. Underwood and Fisher [57] stressed the importance of direct sampling because it can facilitate the detection of invasive species as well as population trends in threatened and bioindicator species. Among all the sampling methods that were used in our study, the pitfall trap yielded the highest number of ant species. Several others studied have showed the efficiency of pitfall in ant sampling [56,57], especially when combined with other methods such as direct sampling [56] or monoliths [37,58].

Baits seemed to be unsuitable for estimating the specific diversity but could be useful for the assessment of ant functional groups [59].

However, the different sampling methods used in this study seemed to be complementary as far as no single method was able to yield all the species identified (43). Only *Ph. tenuinodis* and *T. oscaris* were sampled throughout all the methods. 

The value of the estimator of total observed species (Chao, Jacknife 1 and Bootstrap), were all higher than 40 which was the total number observed species in our study area. This indicates that species richness could be improved with additional sampling effort. 

Myrmicinae were the most abundant not only in species number (22 species) but also in the occurrences. The same pattern was observed by Yeo et al. [37] in a margin of tropical forest at Oumé (Côte d’Ivoire). In their study, the two other most important subfamilies in term of species richness were respectively Ponerinae and Formicinae. This was a little bit different from our results because we found that Formicinae were the second most represented followed by Ponerinae. Gómez and co-authors [60] found similar results. Indeed, Myrmicinae (with 48% of extant ant total species), Formicinae (23%) and Ponerinae (10%) are respectively the three largest subfamilies in the world [61]. Our result is similar to this global pattern. 

#### Specificity of the Two Habitats and Bio Indicator Species

Although the Estimators of total species indicated that less species were sampled than present, the values of Simpson index of diversity (over 0.8) and Shannon Wiener index (over 2.40) showed that both habitats had similar diverse ant populations. This could be because both were derived from the original agricultural land of twenty-years previously.

The habitats shared less than half the overall range of species according to Jaccard (32.5%) and Sorensen classic (49%) suggesting habitat preference by the different ant species. Hence non shared species sampled in the covered area included arboreal ants (*O. longinoda*) and leaf litter dwelling ants (*Strumigenys bernardi* and *S. sistrura*). These are specific to respectively canopy [62,63] or organic rich soils [40] and also habitats such as evergreen forest, forest [37], and shade cocoa plantations [39] and could be useful bio-indicator species. In the open area other species were found like *Brachyponera sennaarensis* a species that prefers human habitats [64,65]. It is also a common species in savannah [66]. The invasive species *Paratrechina longicornis* also known as crazy ant can be an indicator for disturbed habitats [67]). Other species from the open habitats here were *Nylanderia boltoni* and *T. oscaris* which are commonly found in grassland and open woodland [68] and are also suitable as indicators of these landscapes. Similar studies carried out in urban areas showed the same pattern. In the city of Uberlandia (southern Brazil), Pachero and Vasconcelos [69] found that ant species richness was greater in urban forested parks than public and commercial squares. Thus, the presence of vegetation [69] and habitat age [70] are key characteristics for ant conservation in afforested areas [39].

### 4.2. Termites

Seventeen species of termites belonging to four feeding groups were identified. With seven out of these 17 species, the fungus growing termites group was the most important. Termites belonging to this group such as species of *Allodontermes*, *Ancistrotermes*, *Macrotermes*, and *Microtermes* are usually common to rain forest and savannah [71]. Their presence in several habitats types is due to their ability to feed on a large variety of rich cellulose materials such as fallen leaves, herbs, twigs, wood logs [72] even furniture and other wooden structure inside habitations. Grass feeding termites composed the second most important feeding group. All the members of this group belong to a single genus: *Trinervitermes*. As they feed exclusively on green or dry grasses, they are only found in the open area such as savannahs. Termites of the soil feeding group were encountered in all the transects, however species that exclusively dwelt on organic rich soils (*Basidentitermes mactus*), were sampled in the covered area. Indeed, several studies indicated that Cubitermitinae subfamily to which *Basidentitermes* species belong are commonly found in habitats such as tropical rain forest and savanna woodland with a higher concentration of organic maters in the soil [73]. It seems that the soil of the covered area is suitable for the settlement of *Basidentitermes mactus.* Hence as a result of afforestation, the soil of the covered area appears to be more enriched than that of open area. On the other hand, *Adaiphrotermes sp* and *Astalotermes sp* which also belong to the soil feeding group were encountered in both habitats. This repartition could be linked to the vegetation because species belonging to *Astalotermes* are not only encountered in tropical rain forest and savannah woodland but also in semi desert areas [74]. So, they could be encountered in both grass savanna such as the open area of our study.

Both areas shared more than half of total species (10 out of 17) however the repartition of species between the habitats seemed to be linked to the vegetation. Grass feeding termites (*Trinervitermes* species) were almost exclusively encountered in the open area (except the edge of the border transect of the covered area). Also, *Basidentitermes mactus* was exclusively encountered in the covered area. The occurrence of this species in that habitat could be linked to the richness of organic matter in the soil by the decomposition of fallen leaves into litter. The above-mentioned termite species are potential bio-indicators of grassland (*Trinervitermes* species) and of forest and wooded savannah (*Basidentitermes mactus*).

## 5. Conclusions

This study was a contribution to the knowledge of ant fauna and also the first that compared the vegetation of two adjacent habitats and linked this with ants and termites in Togo. Some of the above list of 43 ant species (Table 2) have been recorded for the first time in the country (in comparison to the Antwiki list [74] and the ants checklist of Togo from “Ants of Africa” website [45]). Some of these species (ants as well as termites) shared the two habitats while others seemed to be exclusive to a specific habitat. Some species could be catalogued as potential bioindicators because their presence requires a specific biotic and abiotic quality of the environment. Newly creating such conditions in highly degraded ecosystems like abandoned agricultural lands could reduce the loss of rare and critical species. 

## Figures and Tables

**Table 1:** List of encountered plants species in the study site.

Habitats	Plant Species
Covered area	*Acacia auriculiformis* A.Cunn. ex Benth *(Fabaceae)*
	*Azadirachta indica* (Meliaceae)
	*Blighia sapida* K. D. Koenig (Sapindaceae)
	*Delonix regia* (Boj.ex Hook.) Raf. (Caesalpiniaceae)
	*Ficus sp* (Moraceae)
	*Irvingia gabonensis* (Aubry-Lecomte ex O’Rorke) Baill. (Irvingiaceae)
	*Khaya senegalensis* (Desr.) A. Juss. (Meliaceae)
	*Mangifera indica* L. (Anacardiaceae)
	*Polyalthia longifolia* Sonn. (Annonaceae)
	*Senna Siamea* (Lam.) Irwin et Barneby (Fabaceae)
Open area	*Azadirachta indica* (Meliaceae) *Heteropogon contortus* (L.) P. Beauv. Ex Roem. & Shult (Poaceae)

**Table 2:** The overall taxonomic composition of ants collected in the botanic garden with total number of species occurrences and sampling methods (D (direct sampling)*, ^#^* D (Ants collected only through direct sampling) G (groundnut bait), H (honey bait), M (monolith), P (pitfall), T (tuna bait)). Potential bio-indicator species for *wooded habitats and **grass land habitats.

Sub-Family (Percentage of Species)	Species	Total Occurrences		Methods
		Open Area	Covered Area	
Dolichoderinae (4.65%)	*Tapinoma lugubre* (Santschi, 1917)	8	3	M, P
	*Tapinoma melanocephalum* (Fabricius, 1793)	-	2	P
Dorylinae (2.33%)	*Dorylus* sp.1	1	2	M, P
Formicinae (30.23%)	*Camponotus acvapimensis* (Donisthorpe, 1945)	1	2	P, D
	*Camponotus controversus* (Santschi, 1916)	4	-	M, P, D
	*Camponotus* sp.1	-	-	*D
	*Camponotus maculatus* (Fabricius, 1782)	3	-	M, P, D
	*Camponotus sericeus* (Fabricius, 1798)	1	2	P, D
	*Camponotus vividus* (Smith, F, 1858)	-	-	^#^ D
	*Lepisiota guineensis* (Mayr, 1902)	3	1	M
	*Lepisiota laevis* (Santschi, 1913)	-	1	M, D
	*Lepisiota* sp.1	10	2	M, H, P, D
	*Lepisiota* sp.2	-	1	M, D
	Nylanderia boltoni ** (La Polla & Fisher, 2011)	4	-	M, P
	*Oecophylla longinoda* (Latreille, 1802)	-	1	P, D
	Paratrechina longicornis ** (Latreille, 1802)	1	-	P
Myrmicinae (51.16%)	*Cataulacus traegaordhi* (Santschi, 1914)	-	1	M, D
	*Cardiocondyla emeryi* (Forel,1881)	3	1	M, P
	*Carebara* sp.1	1	-	M
	*Carebara* sp.2	-	1	P
	*Crematogaster* sp.1	-	3	M, D
	*Crematogaster* sp.2	-	2	M, D
	*Monomorium bicolor* (Emery, 1877)	2	-	P
	*Monomorium exiguum* (Forel, 1894)	-	1	P
	*Monomorium* sp.1	2	2	M, P, D
	*Pheidole excellens* (Mayr,1862)	-	-	^#^ D
	*Pheidole impressifrons* (Wasmann, 1905)	3	12	M, P, D
	*Pheidole senilifrons* (Wheeler, 1922)	25	12	G, M, P, D
	*Pheidole tenuinodis* (Mayr, 1901)	36	31	G, M, H, P, T, D
	*Pheidole* sp.1	5	7	M, P, D
	*Pheidole* sp.2	-	1	P
	*Pheidole* sp.3	-	1	P
	Strumigenys bernardi * (Brown, 1960)	-	2	P
	Strumigenys sistrura * (Bolton, 1983)	-	1	P
	*Tetramorium anxium* (Santschi, 1914)	-	1	M
	*Tetramorium sericeiventre* (Emery, 1877)	2	-	P
	*Tetramorium simillimum* (Smith, F., 1851)	-	2	P, M
	*Trichomyrmex oscaris* **(Forel, 1894)	8	-	G, M, H, P, T, D
Ponerinae (9.3%)	Brachyponera sennaarensis ** (Mayr, 1862)	1	-	M
	*Hypoponera* sp.1	-	1	M
	*Hypoponera* sp.2	2	-	M
	*Hypoponera* sp.3	1	-	M
Pseudomyrmicinae (2.33%)	*Tetraponera mocquerysi* (André, 1890)	-	2	P, D

**Table 3:** Estimation of total ant species richness for the two areas.

		Standard Deviation
Observed species (S)	40	
Chao 2	52.54	6.87
Jacknife 1	53.5	2.5
Jacknife 2	53.5	
Bootstrap	46.75	

**Table 4:** Ant diversity index in the two habitats (S = total observed species for each habitat).

Habitats	Overall Occurrence	S	Simpson 1-D	Shannon
Open area	128	24	0.8617	2.48
Covered area	101	29	0.8658	2.633

**Table 5:** Ant Diversity t test.

	Shannon Index (H)	Simpson Index (D)
Open area	2.4798 ± 0.011071	0.13831 ± 0.00037821
Covered area	2.6333 ± 0.017097	0.1342 ± 0.0060072
	*t* = −0.91421, *df* = 206, *p(same)* = 0.36168	*t* = 0.13114, *df* = 204.31, *p(same)* = 0.89579

**Table 6:** Ant shared species (S* = total observed species for each habitat).

Sample	S	Shared Species	Chao Shared Estimates	Jaccard Classic (%)	Sorensen Classic (%)
Open area	24	12	19.2	32.5	49
Covered area	29				

**Table 7:** Overall occurrence of termites for the two habitats. Potential bio-indicator species for * wooded habitats and ** grass land habitats.

Sub-Family	Termites Species	Feeding Group	Occurrence		
			Open Area	Covered Area	Total
Apicotermitinae	*Adaiphrotermes* sp	S	19	7	26
	*Astalotermes* sp	S	8	5	13
Cubitermitinae	Basidentitermes mactus * (Sjöstedt, 1911)	S	0	24	24
Macrotermitinae	*Allodontermes* sp	F	5	24	29
	*Ancistrotermes cavithorax* ( Sjöstedt, 1899)	F	10	8	18
	*Ancistrotremes crucifer* (Sjöstedt, 1897)	F	3	6	9
	*Macrotermes subhyalinus* (Rambur, 1842)	F	16	5	21
	*Microtermes comprehensa* (Silvestri, 1914)	F	11	1	12
	*Microtermes grassei* (Ghidini, 1955)	F	1	9	10
	*Microtermes tumodiensis* (Grassé, 1937)	F	0	2	2
Termitinae	*Amitermes evuncifer* (Silvestri, 1912)	W	2	0	2
	*Microcerotermes* sp.1	W	4	4	8
	*Microcerotermes* sp.2	W	0	13	13
Nasutitermitinae	Trinervitermes geminatus ** (Wasmann, 1897)	G	16	0	16
	Trinertermes oeconomus ** (Trägårdh, 1904)	G	22	1	23
	Trinervitermes togoensis** (Sjöstedt, 1899)	G	24	0	24
	*Trinervitermes* sp **	G	13	0	13
	Total		154	109	263

**Table 8:** Estimation of termite total species richness for **the two areas.**

		Standard Deviation
Observed S	17	
Chao 2	17.96	1.28
Jacknife 1	20.5	0.5
Jacknife 2	20.5	
Bootstrap	18.75	

**Table 9:** Termites diversity index in the two habitats (S = total observed species for each habitat).

Habitat	Overall Occurrence	S	Simpson 1-D	Shannon
Open area	154	14	0.897	2.397
Covered area	109	13	0.863	2.217

**Table 10:** Diversity t test of termites for the two habitats.

	Shannon Index (H)	Simpson Index (D)
Open area	2.3967 ± 0.00244	0.10297 ± 5.06 × 10^−5^
Covered area	2.217 ± 0.00567	0.1366 ± 0.000239
	*t* = 1.9946, df = 197.18, *p*(same) = 0.047459	t = −1.9759, df = 155.18, *p* (same) = 0.049937

**Table 11:** Shared termite species between the two habitats (S = total observed species for each habitat).

Sample	S	Shared Species	Chao Shared Estimates	Jaccard Classic (%)	Sorensen Classic (%)
Open area	14	10	10.67	58.8	74
Covered area	13				
